# Dimethylglycine Supplementation in Reduced Energy Broilers’ Diets Restores Performance by Improving Nutrient Digestibility

**DOI:** 10.3390/ani10050789

**Published:** 2020-05-02

**Authors:** Sofia Chalvatzi, Georgios A. Papadopoulos, Vasilios Tsiouris, Ilias Giannenas, Ioannis T. Karapanagiotidis, Alexandros Theodoridis, Ioanna Georgopoulou, Paschalis D. Fortomaris

**Affiliations:** 1 Laboratory of Animal Husbandry, Faculty of Veterinary Medicine, School of Health Sciences, Aristotle University of Thessaloniki, University Campus, 54124 Thessaloniki, Greece; schalvat@vet.auth.gr (S.C.); fortomap@vet.auth.gr (P.D.F.); 2 Unit of Avian Medicine, Faculty of Veterinary Medicine, School of Health Sciences, Aristotle University of Thessaloniki, Stavrou Voutyra 11, 54627 Thessaloniki, Greece; biltsiou@vet.auth.gr (V.T.); ioannag@vet.auth.gr (I.G.); 3 Laboratory of Animal Nutrition, Faculty of Veterinary Medicine, School of Health Sciences, Aristotle University of Thessaloniki, University Campus, 54124 Thessaloniki, Greece; igiannenas@vet.auth.gr; 4 Aquaculture Laboratory, Department of Ichthyology and Aquatic Environment, University of Thessaly, Fytokou Street, 38446 Volos, Greece; ikarapan@uth.gr; 5 Laboratory of Animal Production Economics, Faculty of Veterinary Medicine, School of Health Sciences, Aristotle University of Thessaloniki, University Campus, 54124 Thessaloniki, Greece; alextheod@vet.auth.gr

**Keywords:** broilers, dimethylgycine, fat digestibility

## Abstract

**Simple Summary:**

Nutritional emulsifiers are used to counteract any negative impact on birds’ performance by improving fat digestibility. Reducing the energy content of broiler diets, which can be achieved by decreasing the quantity of supplemented fat, could contribute to the formulation of diets with a lower production cost. Dimethylgycine (DMG) is a bioactive compound with multiple applications and functions, which has been used as a dietary supplement in the feed of monogastric animals. The aim of the present study was to investigate the effects of DMG supplementation in reduced-energy diets in broilers on performance and nutrient digestibility. It was shown that DMG supplementation in reduced energy broiler diets restored performance indicators to the levels obtained with a standard diet. This effect was probably mediated by the positive effects on the gastrointestinal function of the broilers after DMG supplementation, as evidenced by the improved nutrient digestibility. It is essential for the digestive system in broilers to function optimally, in order to correspond to increased feed consumption and their high production potential. The dietary supplementation of DMG and ingredients with similar emulsifying properties could be used as supportive means for sustainable broiler production.

**Abstract:**

Reducing the energy content of broiler diets could lead to the formulation of diets with reduced production cost. Dimethylgycine (DMG) has been used as a dietary supplement to enhance dietary fat utilization in poultry. The objective of the present study was to investigate the effects of DMG supplementation in reduced energy diets on performance and nutrient digestibility in broiler chickens. Four hundred and eighty day-old broilers were randomly allocated to three dietary treatments: a standard energy diet (PC treatment), a reduced energy diet by 66 kcal/kg (NC treatment) and the reduced energy diet supplemented with 500 mg/kg of DMG (DMG treatment). Fat digestibility was significantly higher in DMG group, compared to PC and NC groups. Intestines and gizzard lesion scores were found to be lower in the DMG group compared to PC. DMG supplementation resulted in lower jejunum pH and ileum viscosity in broilers. Overall, the present study showed that DMG supplementation in reduced energy broiler diets restored growth performance to the levels obtained with a standard diet. This result was probably mediated by the positive effects on the gastrointestinal function of the broilers after DMG supplementation, as evidenced by the improved nutrient digestibility, the reduced gross lesion scores and the lower values in intestinal pH and viscosity.

## 1. Introduction

Dietary fat is the most energy dense nutrient, and its efficient utilization is a prerequisite for improved performance [1]. In young birds, however, the capacity to digest and absorb dietary fat is restricted [2]. This limitation can be overcome by adding emulsifiers into diets. Emulsifying agents increase the active surface of fat, promoting lipase activity and assisting in the formation of micelles [3]. Emulsifiers like soy lecithin [4] and lysolecithin [5,6] have been tested in poultry diets with promising results. Dimethylglycine (DMG) is a tertiary amine formed in liver mitochondria during the remethylation pathway of homocysteine to methionine by the removal of one methyl group from betaine [7]. DMG exerts an emulsifying action, and has been used effectively in broilers [8,9,10,11] and also in pigs [12,13]. Studies conducted in both animal species have shown that DMG dietary supplementation can improve the digestibility of fat [11,12], protein [8,11,12] and nitrogen-free extract [8,12], as well as the digestibility of dry matter [8,11], organic matter and a-tocopheryl-acetate [11]. The dosages tested and proven to be beneficial in terms of nutrient digestibility were 167mg/kg [8] and 1000mg/kg [11] of diet in broilers and 500 mg/kg of diet in sows [12]. Nevertheless, as regards broilers, no performance effect was observed at the dosage of 167 mg/kg. On the contrary, positive effects on final body weights [10], production value [9,10] and feed conversion ratio [10] were recorded after broiler diet supplementation with the highest approved dosage, that is, 1000 mg/kg of diet. Moreover, the protective action of DMG against hepatic, intestinal and gastric damage has been demonstrated in trials conducted with piglets [13], mice [14] and rats [15].

Based on the emulsifying potential of DMG and its capacity to promote the digestibility of other nutrients, it could be hypothesized that it would positively affect broilers’ performance when fed diets with a reduced energy level. Therefore, the present study investigated whether dietary supplementation with 500 mg DMG/kg of diet, that is, half the maximum approved and proven beneficial dosage, in a reduced energy diet throughout the broilers’ growth cycle could aid in recovery of performance to levels obtained with a standard diet. Moreover, considering the potential of DMG to attenuate hepatic, intestinal and gastric damage, and the lack of previous published data regarding broilers, the existence of gross lesions in the gastrointestinal tract, as well as alterations of pH and viscosity, were also evaluated.

## 2. Materials and Methods

### 2.1. Ethical Statement

The experimental procedures were in line with all welfare requirements described by Good Farming Practice Guidelines (Directive 2010/63/EC; Commission recommendation 2007/526/EC) and approved by the Ethical Committee branch of the Research Committee of Aristotle University of Thessaloniki in Greece.

### 2.2. Broilers, Experimental Design and Diets

A total of 480 broiler chicks from Ross 308, at the age of 1 day from hatching, obtained from a commercial hatchery, were raised until the 42nd day of age. Upon arrival, birds were randomly allocated to 3 dietary treatments with 8 replicate pens of 20 chickens per group of treatments. The first group was assigned as the positive control (PC treatment) and fed a standard caloric diet formulated to meet nutritional requirements. The second group received a diet with energy reduced by 66 kcal/kg, and was designated as the negative control (NC treatment). Energy reduction was performed by removing approximately 1% of the soybean oil from the standard caloric diet. Necessary modifications in the inclusion rates of soft wheat and soybean meal were also applied in the NC diet so as to ensure compliance with nutrient specifications. The third group was fed the diet of the NC treatment, supplemented with 500 mg DMG (Taminizer (^®^) D; Taminco N.V., Ghent, Belgium) /kg of diet (DMG treatment). This additive contained at least 97% DMG-Na. The ingredients and the calculated analysis of the diets used in the 3 dietary treatments are shown in Table 1.

### 2.3. Performance Evaluation

The performance parameters recorded throughout the experimental period included body weight, weight gain, feed intake and feed efficiency. The body was digitally weighed every two weeks of each feeding phase, from the initial to the final body weight (1 d, 14 d, 28 d, and 42 d). Weight gains, feed intakes and feed conversion ratios on a pen basis were determined for the respective growth phases.

### 2.4. Nutrient Digestibility, Chemical Analysis and Calculations

Titanium dioxide (TiO_2_; E171 titanium dioxide, IMCD Benelux N.V., Mechelen, Belgium) was incorporated as a digestibility marker in the experimental diets of the broilers at a concentration of 0.3%. Diets were offered to birds from day 29 for 5 days, while excreta collection on plastic mats took place on days 33 and 34. Feed and excreta content of moisture, nitrogen and fat was determined according to the methods of the Association of Official Analytical Chemists [16]. Gross energy was calculated using an adiabatic bomb calorimeter (IKA^®^ Calorimeter System C 5000 Control, IKA^®^-Werke GmbH & Co. KG, Staufen, Germany). Determination of titanium dioxide was performed by inductively coupled plasma optical emission spectrometry following the procedure of van Bussel et al. [17]. Samples of feces and feedstuff were acid digested by using appropriate amounts of concentrated HNO_3_ and concentrated H_2_SO_4_ in hood. An accurately weighed portion of the sample, ca. 100–200 mg, was placed into the vessel, with the subsequent addition of the acids. After heating, the digest was diluted to volume with deionized water. The final solutions were analyzed by ICP-AES against acidified aqueous and matrix matched standards. All glassware and digestion vessels were soaked in freshly prepared 10% v/v HNO_3_ all night and finally washed three times with Milli-Q quality water. An axial viewing plasma spectrometer model Perkin Elmer Optima 3100 XL (Perkin Elmer Inc., Waltham, MA, USA) was used.

Digestibility was calculated according to the equation

(1) $$\%\mathrm{Digestibility}=\left(1-\frac{{\mathrm{marker}}_{\mathrm{feed}\left(\%\right)}\times {\mathrm{nutrient}}_{\mathrm{excreta}\left(\%\right)}}{{\mathrm{marker}}_{\mathrm{excreta}\left(\%\right)}\times {\mathrm{nutient}}_{\mathrm{feed}\left(\%\right)}}\right)\times 100$$

Nitrogen corrected AMEn value was calculated using the equation

(2) $${\mathrm{AMEn}}_{\mathrm{kcal}/\mathrm{kg}}={\mathrm{GE}}_{\mathrm{feed}\;\mathrm{kcal}/\mathrm{kg}}-[\frac{{\mathrm{GE}}_{\mathrm{excreta}\;\mathrm{kcal}/\mathrm{kg}}\times {\mathrm{marker}}_{\mathrm{feed}\%}}{{\mathrm{marker}}_{\mathrm{excreta}\%}}-8.22\times \left(N_{\mathrm{feed}\%}-\frac{N_{\mathrm{excreta}\%}\times {\mathrm{marker}}_{\mathrm{feed}\%}}{{\mathrm{marker}}_{\mathrm{excreta}\%}}\right)]$$

where GE = gross energy, N = nitrogen and 8.22 = is the energy equivalent of uric acid nitrogen (8.22 kcal/kg uric acid nitrogen).

### 2.5. Evaluation of Gross Lesions in Gastrointestinal Tract Organs, Intestinal pH and Viscosity

At the end of each feeding phase (days 14, 28 and 42) one healthy bird from each floor pen (8 birds per treatment) was randomly chosen and euthanized in the Avian Unit of the Faculty of Veterinary Medicine. The gastrointestinal tract was removed immediately and divided into its anatomical parts (gizzard, duodenum, jejunum, ileum, caeca). The intestine, gizzard and liver were macroscopically examined and scored for gross lesions. The intestines were macroscopically examined and scored according to a zero to four scoring system as follows: 1 = normal intestinal mucosa; 2 = hyperaemia of intestinal mucosa and/or undigested intestinal content in the last third of intestine; 3 = congestion and thickness of intestinal mucosa and/or watery intestinal content; 4 = haemorrhages of intestinal mucosa. The gizzards were macroscopically examined and scored for gross lesions using a zero to two scale, as described by Novoa-Garrido et al. [18]. In particular, the gross gizzard lesions received a zero score in case they were absent, a score one if erosion of cuticula of gizzard was observed and a score two if ulcers of the cuticula of the gizzard were present. The livers were macroscopically examined and scored for gross lesions using a zero to two scale, as described elsewhere [19]. The zero score corresponded to normal liver, gall bladder and bile, the score one to congestion of liver and/or thickness and distention of gall bladder and/or discoloration of bile, and the score two to necrotic lesions of the liver.

At 28 and 42 days of age, the digesta of the duodenum, jejunum, ileum and caecum from each bird were immediately collected after euthanasia in separate tubes, and vortexed to obtain a homogenous content from each anatomical part of the intestine per bird. The pH of the duodenum, jejunum, ileum and caecum from each bird was measured using a digital pH-meter (pH 315i, WTW Wissenschaftlich- TechnischeWerkstätten, Weilheim, Germany). The digesta of the jejunum and ileum were used to measure viscosity. The viscosity of intestinal digesta was determined according to Tsiouris et al. [19] using a Brookfield DV-II + PRO Digital Viscometer (Brookfield Engineering Laboratories, Stoughton, MA, USA). Two readings were taken from each sample and were represented in units of centipoise (CP).

### 2.6. Statistical Analysis

Data were analyzed using the Statistical Package for the Social Sciences (SPSS) software (SPSS 25.0 Version, Chicago, IL, USA). Statistical significance was considered at *p* ≤ 0.05. Treatments (PC, NC, DMG) were regarded as fixed factors during statistical analysis. For the investigated parameters (performance, nutrient digestibility, gross lesions, viscosity and pH), the pen was the experimental unit (8 per treatment). Results are presented as mean ± standard deviation (SD). The effect of treatments on performance, digestibility and viscosity variables was analyzed with one-way ANOVA of GLM procedure of SPSS, and post-hoc comparisons between treatments were made by Duncan’s test.

## 3. Results

### 3.1. Performance

The effect of dietary supplementation with DMG on broilers’ performance is shown in Table 2. Birds fed the negative control diet had a significantly lower mean body weight at the age of 42 days compared to birds in the positive control group. Their body weight gain for the 29–42 days and the total feeding periods was also significantly lower. On the contrary, birds fed the negative control diet supplemented with DMG had mean final body weights and mean total body weight gains that did not differ from those of the positive control group birds. The feed intake of birds receiving DMG was significantly higher compared to that of the negative control group only for the 29–42 day period. Feed conversion ratio did not differ among treatment groups.

### 3.2. Nutrient Digestibility

The effect of dietary supplementation with DMG on nutrient digestibility is shown in Table 3. Statistically significant differences were not observed regarding the dry matter digestibility. Birds receiving the negative control diet supplemented with DMG exerted an improved crude protein digestibility compared to birds in the negative control group. The crude fat digestibility was also enhanced in birds in the DMG group compared to birds in the positive and negative control groups. Furthermore, AMEn of the negative control diet was lower than AMEn of the positive control diet. However, AMEn of diets supplemented with the tested product did not differ from that of the positive control group.

### 3.3. Gross Lesions in Gastrointestinal Tract Organs

The mean grades of lesion scores in the intestine, liver and gizzard of birds in each experimental group at different ages are given in Table 4. Although no statistically significant differences among treatments were observed in intestines on day 14, the mean lesion score on days 28 and 42 were statistically lower in birds supplemented with DMG compared to the PC group. No lesions were observed in the livers of all birds at the ages of 14 and 42 days. On day 28, lesions were detected only in the negative control group. Lesion scores in gizzards did not differ among the treatment groups on the 14^th^ day. On days 28 and 42, the lesion scores in the DMG treatment were lower compared to the negative and the positive control groups, respectively.

### 3.4. Intestinal pH and Viscosity

The average values of intestinal pH and viscosity estimated at the 28^th^ and 42^nd^ day of age are shown in Table 5. Regarding pH values, most differences between treatments were found at the 28^th^ of age. At the jejunum, pH was significantly lower for both the NC and DMG group compared to the PC, while at the ileum, pH was significantly higher in the DMG group than PC and NC. At the caeca, pH was lower in the DMG than NC, while the PC had intermediate values. At 42 days of age, pH at the jejunum was lower in the DMG compared to NC.

No significant difference was found for viscosity values at 28 days of age. At 42 days, viscosity in the ileum was significantly lower in the PC and DMG compared to NC.

## 4. Discussion

The digestive tract in broilers needs to function at an optimal level, because of the increased feed intake and the high production rate [20]. Therefore, nutrient digestibility should also be estimated when evaluating the effects of various feed ingredients or additives [20]. In the present study, we evaluated the effects of DMG on broilers’ performance and nutrient digestibility. Despite the energy reduction of the tested diet, which was applied at all growth stages, supplementation of DMG resulted in equal performance to that of the PC group. These results confirmed our initial hypothesis that DMG, by acting as an emulsifying agent, can improve nutrient digestibility and compensate for an energy reduction in broilers’ diets. It was previously reported that DMG supplementation, apart from increasing fat digestibility, improved the apparent faecal digestibility of the crude protein and carbohydrate fraction in broilers [8,11]. Consumption of the NC diet resulted in inferior broiler performance in terms of final body weight and body weight gain. Thus, it could be suggested that this gap in performance was ameliorated by DMG supplementation at a rate of 500 mg/kg to such an extent that the performance of the DMG group was equal to that of the positive control group.

To our knowledge, this is the first report that 500 mg DMG/kg of diet can improve the final body weight and body weight gain in broilers. Elsewhere, the beneficial effect of a higher dose of 1000mg/kg of diet on final body weights [10], production value [9,10] and feed conversion ratio [10] in broilers has been reported. On the contrary, no influence on performance traits was recorded either by Prola et al. [11] for the dose of 1000mg/kg or by Kalmar et al. [8] for a much lower dose of 167 mg/kg. In piglets, 0.1% Na-DMG has been shown to be effective in ameliorating the detrimental effects of low birth weight on growth performance by improving body weight gain, feed intake and feed efficiency [13].

The observed improvement in performance in our trial is in accordance with enhancement both in crude fat and crude protein digestibility, and in AMEn. In line with our results, Cools et al. [12] demonstrated that DMG addition in the diets of sows at the same rate resulted in an improved apparent digestibility coefficient for crude fat and crude protein. The effects of DMG at a rate of 500g/kg have not been previously studied in broilers. However, the apparent faecal digestibility of crude protein and nitrogen-free extract was improved after supplementation into broilers diets of 167 mg Na-DMG/kg of diet in a trial by Kalmar et al. [8]. Prola et al. [11] also demonstrated that the addition of 1000mg Na-DMG/kg to the diets of broilers during the growing phase improved the apparent digestibility of dry matter, organic matter, crude protein and a-tocopheryl-acetate in low and high fat diets; fat digestibility was improved only in low fat diets.

Driven by strong indications that DMG can contribute as a protective agent against oxidative stress induced damages in organs like the intestine, liver and stomach [13,14,15], we evaluated gross lesions in these organs. DMG supplementation resulted in lower intestinal lesion scores at different days of age, especially compared to the PC treatment. This effect could be attributed to several factors. It can be hypothesized that, due to an improvement in nutrient digestion, less substrate was available for bacterial growth and hence for an increase in lesion scoring. In general, intestinal disease conditions damage intestinal epithelium and negatively affect the absorption of nutrients [1]. Moreover, Bai et al. [14] have shown that DMG ameliorated oxidative damage in the intestine by upregulating the expression of antioxidant-associated genes and alleviating mitochondrial dysfunction. Similarly, in rats, the anti-ulcer activity of dimethylglycine due to free radical scavenging activity and the protection of gastric mucosa has been shown [15]. In the current study, pH at the jejunum was lower in the DMG-supplemented group compared to the PC both at 28 and 42 days of age. It is plausible that this lower pH level could have also contributed to the lower intestinal scoring index. To our knowledge, such an effect of DMG on intestinal pH has not been reported previously. The jejunum is the major site of the digestion and absorption of fat in poultry [21]. Thus, the decreased pH levels at the jejunum site may be related to the improved fat utilization in the DMG group. On the other hand, the impaired nutrient digestibility in the NC group occurred together with increased pH at the jejunum and increased viscosity at the ileum at 42 days of age. It is suggested that increased intestinal viscosity depresses nutrient digestibility and subsequently performance [22], both of which occurred in the NC group. This field warrants further investigation.

The gizzards of birds that received the NC diet exhibited a higher lesion score on day 28, but a lower score at the age of 42 days, compared to birds of the PC groups. Nevertheless, gizzards of birds supplemented with DMG had a similar or even lower lesion score than birds in the positive control group. Fat emulsification is initiated in the gizzard, due to bile salts and monoglycerides from digesta refluxed from the duodenum [1]. A coarse emulsion of lipids is formed by the acidic environment, the peptic digests of protein and the mechanical activity of the gizzard [1]. It could be hypothesized that this first stage of fat emulsification at gizzard level was further promoted by the action of DMG, and may have contributed to a lower lesion score. The gastroprotective activity of DMG has been demonstrated in rats by Hariganesh and Prathida [15]. The authors attributed the recorded anti-ulcer effect to its free radical scavenging activity and cytoprotection of gastric mucosa.

## 5. Conclusions

Dietary supplementation of DMG in broilers improved crude fat and protein digestibility as well as AMEn. Furthermore, it decreased lesion scoring in different parts of the intestine and in the gizzard. Overall, DMG supplementation is suggested as a dietary intervention towards efficient and sustainable broiler production.

## Figures and Tables

**Table 1:** Ingredient and nutrient composition of the experimental diets according to the stage of growth (on dry matter basis).

Items	Starter (1–14 d)		Grower (15–28 d)		Finisher (29–42 d)	
	PC ^a^	NC ^b^	PC	NC	PC	NC
Ingredients (g/kg)						
Wheat soft	414.7	429.9	356.7	371.5	410.9	425.9
Soybean meal 47%	296.2	292.4	303.4	299.7	247.8	244.1
Maize	200.0	200.0	250.0	250.0	250.0	250.0
Fish meal	20.0	20.0	-	-	-	-
Limestone	14.3	14.3	14.1	14.1	13.4	13.4
Soybean oil	25.4	14.0	48.4	37.1	51.1	39.7
Monocalcium phoshate	13.8	13.8	13.5	13.5	12.9	12.9
Premix ^c^	5.0	5.0	5.0	5.0	5.0	5.0
DL—methionine	3.2	3.2	2.5	2.5	2.1	2.1
L—Lysine	2.9	3.0	2.0	2.1	2.2	2.3
Sodium chloride	2.1	2.0	2.3	2.3	2.2	2.2
L—threonine	1.3	1.3	0.7	0.7	0.8	0.8
Sodium bicarbonate	1.1	1.1	1.4	1.5	1.6	1.6
Nutrient composition ^d^						
Crude protein (g/kg)	220.0	220.0	205.0	205.0	185.0	185.0
Ether extract (g/kg)	45.0	33.8	67.4	56.2	69.6	58.4
AMEn (kcal/kg)	2970	2904	3109	3043	3169	3103
Crude fibre (g/kg)	25.9	26.1	26	26.2	25.1	25.3
Dig. Lysine (g/kg)	12.1	12.1	10.6	10.6	9.4	9.4

^a^ PC: positive control diet providing energy based on recommendations. ^b^ NC: negative control diet with a nitrogen-corrected apparent metabolizable energy (AMEn) reduced by 66 kcal/kg compared to the positive control diet. ^c^ Provided per kg diet: retinyl acetate, 4.2 mg; cholecalciferol, 0.125 mg; αtocopherol acetate, 50 mg; menadione, 7.0 mg; cobalamin, 0.025 mg; folic acid, 2.0 mg; choline, 750 mg; calcium pantothenate, 20.0 mg; riboflavin, 8.0 mg; niacin, 60.0 mg; thiamin, 6.0 mg; D-biotin, 0.2 mg; pyridoxine, 5.0 mg; Mn (from manganese sulphate), 120 mg; Zn (from zinc sulphate), 100 mg; Fe (from ferrous sulphate), 40 mg; Cu (from copper sulphate), 20 mg; I (from calcium iodate), 1.0 mg; Se (from sodium selenite), 0.3 mg. ^d^ Calculated value. The DMG diets consisted of the NC diet supplemented with 500 mg DMG/kg of diet.

**Table 2:** Performance of broilers in each experimental group, measured at different growth stages.

Parameter	Period	Treatments			*p*-Value
		PC	NC	DMG	
Body weight (g)	1 d	47.9 ± 0.83	47.8 ± 0.72	48.4 ± 0.44	0.211
	14 d	351.6 ± 27.63	334. 1 ± 20.98	361.4 ± 17.88	0.118
	28 d	1365.7 ± 59.75	1290.2 ± 87.45	1345.4 ± 58.87	0.106
	42 d	2544.5 ± 104.79 ^a^	2392. 3 ± 87.60 ^b^	2533.0 ± 74.76 ^a^	0.004
Body weight gain (g)	1–14 d	303.6 ± 27.27	286.2 ± 21.06	313.0 ± 17.91	0.075
	15–28 d	1014.1 ± 34.51	956.1 ± 68.54	984.0 ± 50.18	0.115
	29–42 d	1178.8 ± 67.84 ^a^	1102.1 ± 64.63 ^b^	1187.6 ± 57.59 ^a^	0.026
	1–42 d	2496.6 ± 104.81 ^a^	2344.4 ± 87.44 ^b^	2484.6 ± 74.76 ^a^	0.004
Feed intake (g)	1–14 d	454.4 ± 25.44	447.2 ± 23.69	470.1 ± 31.72	0.251
	15–28 d	1558.9 ± 96.20	1548.7 ± 111.71	1577.0 ± 69.78	0.832
	29–42 d	2171.8 ± 86.10 ^a,b^	2106.1 ± 112.06 ^b^	2231.3 ± 61.51 ^a^	0.035
	1–42 d	4185.2 ± 151.42	4102.0 ± 227.76	4278.4 ± 109.16	0.141
Feed conversion ratio (g:g)	1–14 d	1.50 ± 0.08	1.57 ± 0.09	1.50 ± 0.03	0.126
	15–28 d	1.54 ± 0.10	1.63 ± 0.14	1.61 ± 0.11	0.328
	29–42 d	1.84 ± 0.06	1.92 ± 0.15	1.88 ± 0.07	0.383
	1–42 d	1.68 ± 0.06	1.75 ± 0.09	1.72 ± 0.05	0.136

Mean ± standard deviation; ^a,b^ mean values in the same row with a different superscript differ significantly (*p* ≤ 0.05).

**Table 3:** Mean values of nutrient digestibility in each experimental group.

Parameter	Treatments			*p*-Value
	PC	NC	DMG	
Dry matter digestibility (%)	80.8 ± 3.38	77.9 ± 2.26	80.9 ± 3.78	0.400
Crude protein digestibility (%)	72.6 ± 4.77 ^a,b^	69.3 ± 3.68 ^b^	74.4 ± 2.67 ^a^	0.046
Crude fat digestibility (%)	77.6 ± 4.15 ^b^	80.1 ± 5.82 ^b^	85.5 ± 4.57 ^a^	0.014
AMEn (kcal/kg)	3079 ± 65.88 ^a^	2970 ± 65.25 ^b^	3049 ± 90.16 ^a^	0.023

Mean ± standard deviation; ^a,b^ Mean values in the same row with a different superscript differ significantly (*p* ≤ 0.05).

**Table 4:** Mean grade of lesion scores in the intestine, liver and gizzard in each experimental group.

Organ	Age	Treatments			*p*-Value
		PC	NC	DMG	
Intestine	14	1.70 ± 0.68	1.50 ± 0.53	1.40 ± 0.52	0.505
	28	2.80 ± 0.42 ^a^	2.30 ± 0.68 ^b^	1.90 ± 0.32 ^b^	0.002
	42	1.70 ± 0.68 ^a^	1.40 ± 0.52 ^a,b^	1.00 ± 0.00 ^b^	0.013
Liver	14	0.00 ± 0.00	0.00 ± 0.00	0.00 ± 0.00	-
	28	0.00 ± 0.00 ^b^	0.70 ± 0.95 ^a^	0.00 ± 0.00 ^b^	0.010
	42	0.00 ± 0.00	0.00 ± 0.00	0.00 ± 0.00	-
Gizzard	14	1.00 ± 0.82	1.20 ± 0.63	1.00 ± 0.67	0.769
	28	0.30 ± 0.48 ^b^	1.00 ± 0.82 ^a^	0.10 ± 0.32 ^b^	0.004
	42	0.60 ± 0.52 ^a^	0.20 ± 0.42 ^b^	0.10 ±0.32 ^b^	0.034

Mean ± standard deviation; ^a, b^ mean values in the same row with a different superscript differ significantly (*p* ≤ 0.05). For mean value estimation, eight samples per treatment were considered. Intestinal gross lesions scoring system: 1 = normal intestinal mucosa; 2 = hyperaemia of intestinal mucosa and/or undigested intestinal content in the last third of the intestine; 3 = congestion and thickness of intestinal mucosa and/or watery intestinal content; 4 = haemorrhages of intestinal mucosa. Gizzard gross lesions scoring system: 0 = normal gizzard; 1 = erosion of cuticula of gizzard; 2 = ulcers of cuticula of gizzard. Liver gross lesions scoring system: 0 = normal liver, gall bladder and bile; 1 = congestion of liver and/or thickness and distention of gall bladder and/or discoloration of bile; 2 = necrotic lesions of liver.

**Table 5:** Average values of pH and viscosity at different parts of the intestine, measured at 28 and 42 days of age.

Parameter	Age	Treatments			*p*-Value
		PC	NC	DMG	
pH duodenum	28	6.82 ± 0.09	6.68 ± 0.21	6.72 ± 0.12	0.125
pH duodenum	42	6.26 ± 0.25	6.34 ± 0.17	6.34 ± 0.13	0.595
pH jejunum	28	6.65 ± 0.11 ^a^	6.37 ± 0.09 ^b^	6.38 ± 0.09 ^b^	<0.001
pH jejunum	42	6.24 ± 0.11 ^ab^	6.34 ± 0.18 ^a^	6.16 ± 0.15 ^b^	0.043
pH ileum	28	6.73 ± 0.16 ^b^	6.89 ± 0.59 ^b^	7.53 ± 0.44 ^a^	0.001
pH ileum	42	7.06 ± 1.17	7.34 ± 0.62	6.66 ± 0,66	0.228
pH caecum	28	7.03 ± 0.85 ^ab^	7.26 ± 0.52 ^a^	6.48 ± 0.34 ^b^	0.025
pH caecum	42	7.03 ± 0.46	7.24 ± 0.47	7.15 ± 0.46	0.584
Viscosity jejunum	28	2.41 ± 0.40	2.07 ± 0.37	2.28 ± 0.28	0.172
Viscosity jejunum	42	1.80 ± 0.54	1.74 ± 0.45	1.46 ± 0.25	0.200
Viscosity ileum	28	2.57 ± 0.92	3.82 ± 1.05	3.45 ± 0.96	0.487
Viscosity ileum	42	2.64 ± 0.47 ^b^	3.80 ± 1.08 ^a^	2.81 ± 0.60 ^b^	0.011

Mean ± standard deviation; ^a, b^ mean values in the same row with a different superscript differ significantly (*p* ≤ 0.05). For mean value estimation, eight samples per treatment were considered.
